# Supplementary material for: A concerted ATPase cycle of the protein transporter AAA-ATPase Bcs1
Source: Nat Commun. 2023 Oct 11;14:6369. doi: 10.1038/s41467-023-41806-5 (PMC10567702; doi:10.1038/s41467-023-41806-5)
Supplement: Supplementary file 1 — Supplementary information [file 41467_2023_41806_MOESM1_ESM.pdf]

A concerted ATPase cycle of the protein transporter AAA-ATPase Bcs1

Yangang Pan[1], Jingyu Zhan[3], Yining Jiang[1], Di Xia[3], Simon Scheuring[1,2,4,*]

[1] Department of Anesthesiology, Weill Cornell Medical College, 1300 York Ave, New York, NY 10065, USA
[2] Department of Physiology & Biophysics, Weill Cornell Medical College, 1300 York Ave, New York, NY 10065, USA
[3] Laboratory of Cell Biology, National Cancer Institute, National Institutes of Health, Bethesda, MD, USA.
[4] Kavli Institute at Cornell for Nanoscale Science, Cornell University, Ithaca, New York 14853, USA
[*] Correspondence should be addressed to: sis2019@med.cornell.edu

SUPPLEMENTARY INFORMATION

Supplementary Figure 1)

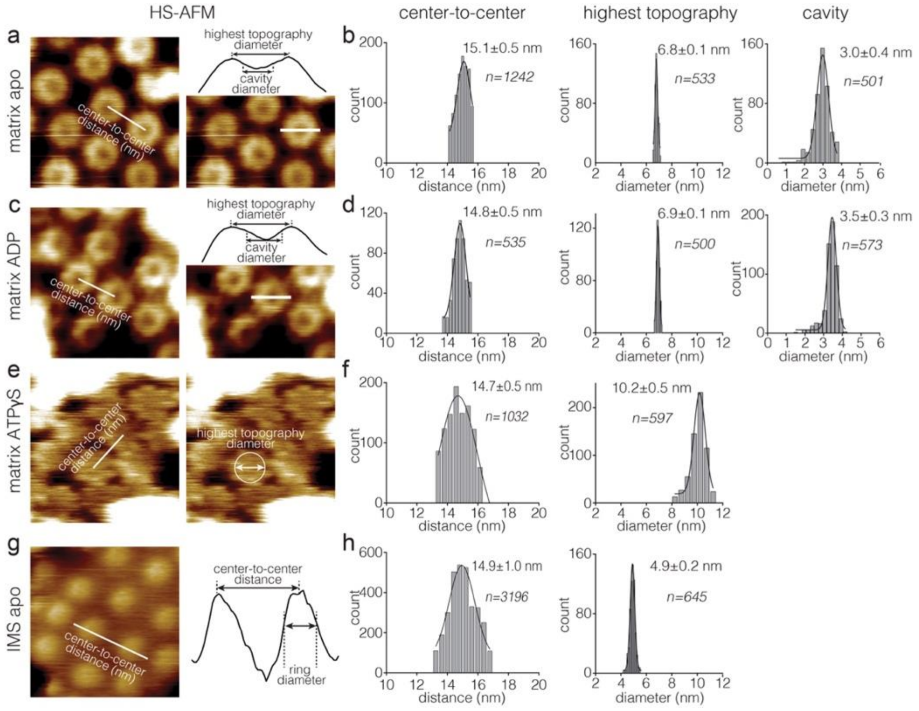

Supplementary Figure 1 | Center-to-center distances (outer diameter) and highest topography diameter of apo, ADP and ATPγS conformations in HS-AFM. a) HS-AFM image of the center-to-center distance measurement, hightest topography diameter and cavity diameter measurements (*left*) of Bcs1 apo conformation exposing the matrix face. **b**) Center-to-center distance distribution, highest topography diameter and cavity diameter histograms of apo conformation Bcs1. **c)** and **d), e)** and **f)**, and **g)** and **h)** Analysis as in a) and b) but for matrix ADP (c,d), matrix ATPγS (e,f), and IMS apo (g,h) conditions. (lines: Gaussian fits, distribution peak positions and number of measurements are indicated in the panels). Similar results as in (a), (c), (e) and (g) were observed in at least 3 independent biological experiments. Source data are provided as a source data file.

Supplementary Figure 2)

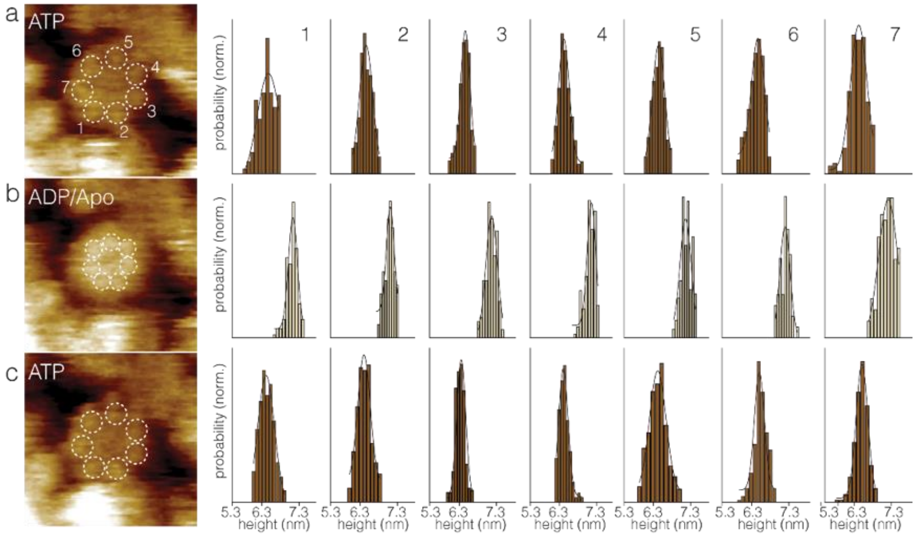

Supplementary Figure 2 | Height distribution analysis of subunits in HS-AFM images. **a)**, **b)** and **c)** Left: High-resolution HS-AFM images of matrix surfaces in ATP (a,c) and apo/ADP (b) conformation (see Figure 2d in main text). Right: Corresponding pixel height histograms of all pixels in the white dashed circles in the left images. Top and bottom: *n* = 256 for each histogram, middle: *n* = 156 for each histogram. Similar results as in (**a**), (**b**) and (**c**) were observed in a minimum of 10 independent biological experiments. Source data are provided as a source data file.

Supplementary Figure 3)

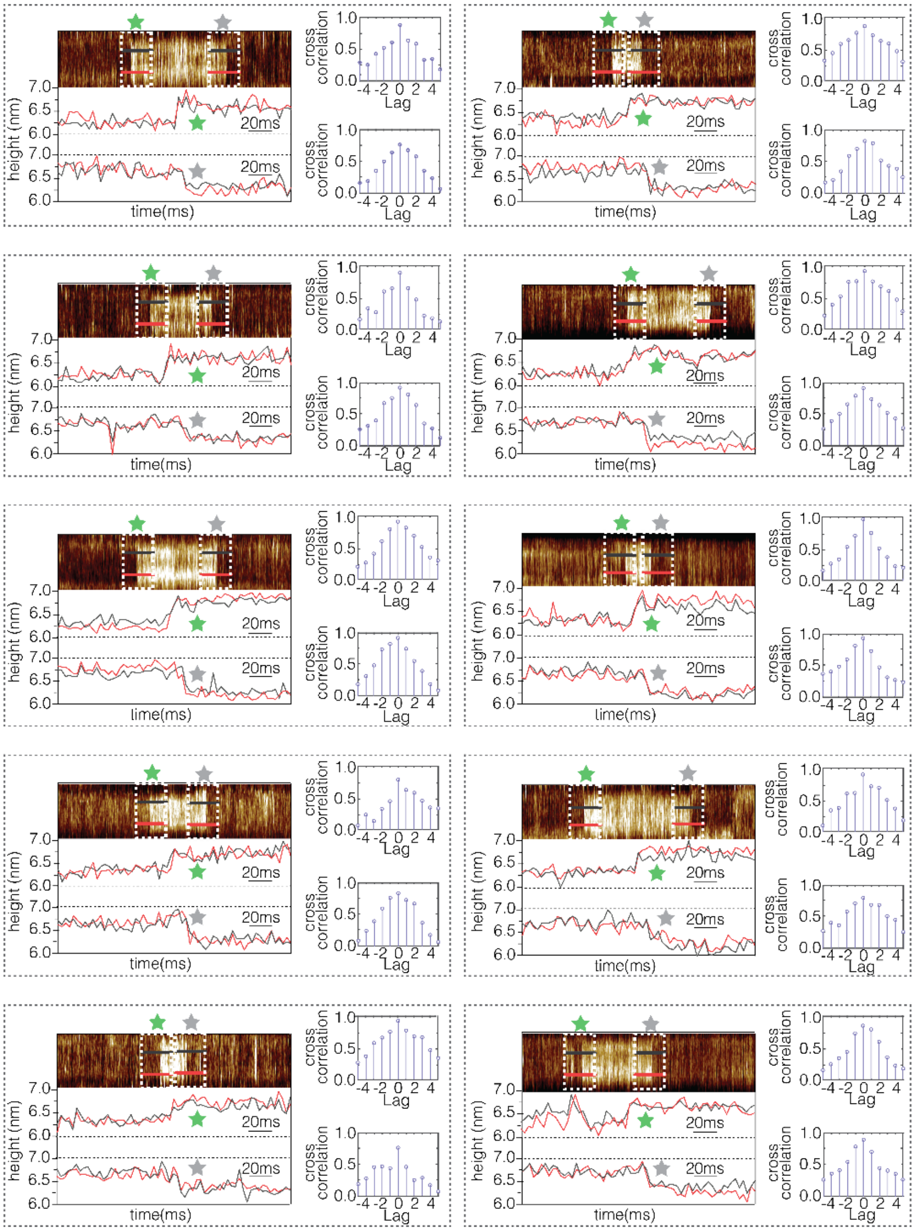

Supplementary Figure 3 | Time-lag cross-correlation analysis of ATP- to apo/ADP- and apo/ADP- to ATP-conformation conformational changes. HS-AFM-LS kymograph and height/time traces of single Bcs1 ring conformational changes (*left*). Green and grey stars indicate the time points of ATP-hydrolysis and ATP-binding related conformational changes, respectively. The time-lag cross-correlation peak analyses (*right*) indicate the time when the two traces are most synchronized (1 lag = 3.3 ms, *i.e.*, the HS-AFM-LS scanning rate). All events peak at a time lag = 0.

Supplementary Figure 4)

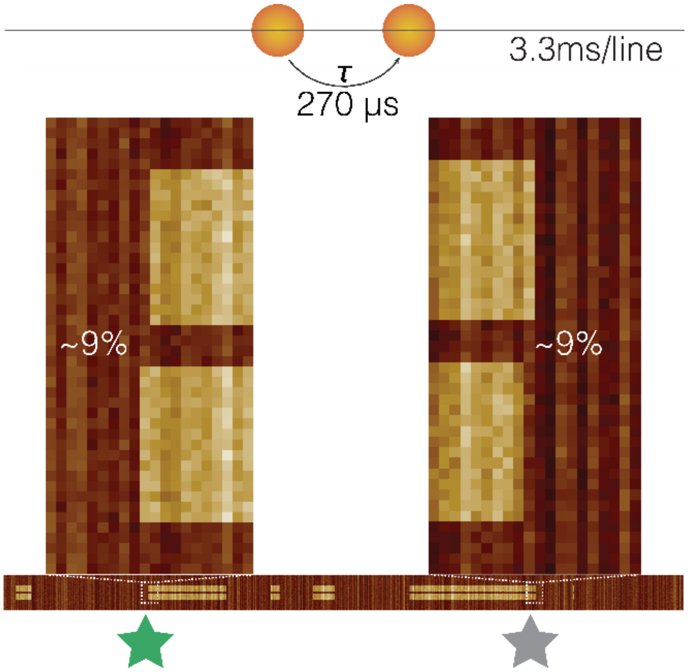

Supplementary Figure 4 | Numerical simulation of the detection probability of non-synchronized height/time traces in HS-AFM-LS experiments at 3.3 ms/line and 270 µs time-lag between peripheral protomers. A 9% probability of detecting time-lag in kymographs recorded at line rate of 3.3 ms/line and 270 µs/across-Bcs1 would be detected at a transition event duration of 270 µs or longer. In our simulations, the distance between the two detection positions and the total line scan size were set to the same values as in the HS-AFM-LS experiments.

Supplementary Figure 5)

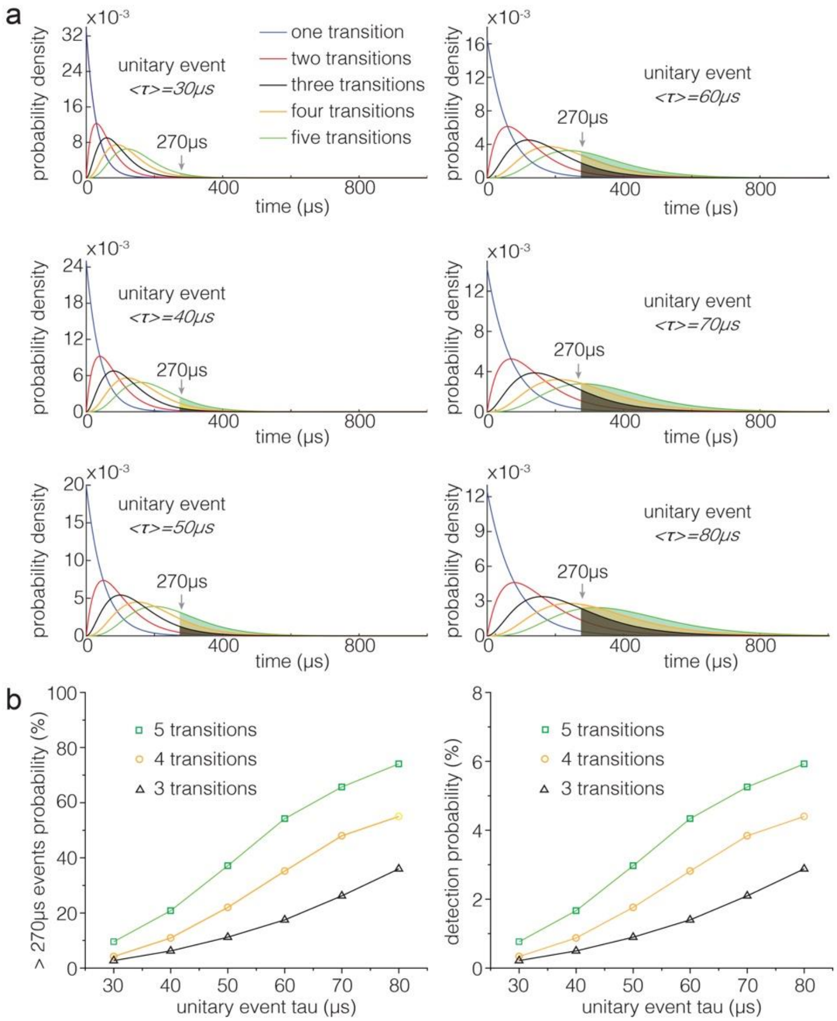

Supplementary Figure 5 | Estimation of Bcs1 transition times at various sequential transition unitary event values. a) Convolution of probability distributions for 1 (blue), 2 (red), 3 (black), 4 (orange) and 5 (green) transitions at various unitary characteristic time constants τ, ranging from 30 μs to 80 μs. The blue line represents the unitary transition event described by a single exponential distribution. The black, orange and green shaded areas represent the events longer than 270 μs for 3, 4 and 5 transitions, respectively. All distributions are theoretical curves plotted using *Eq.* (3) using unitary event times as indicated. **b)** Left: The probability of events longer than 270 μs for a series of unitary time constants values τ ranging from 30 μs to 80 μs. Right: The corresponding theoretical detecting probability in HS-AFM-LS experiments. Black triangles, orange circles and green squares are the probability for 3, 4 and 5 transitions, respectively.

50 **Supplementary Figure 6)**

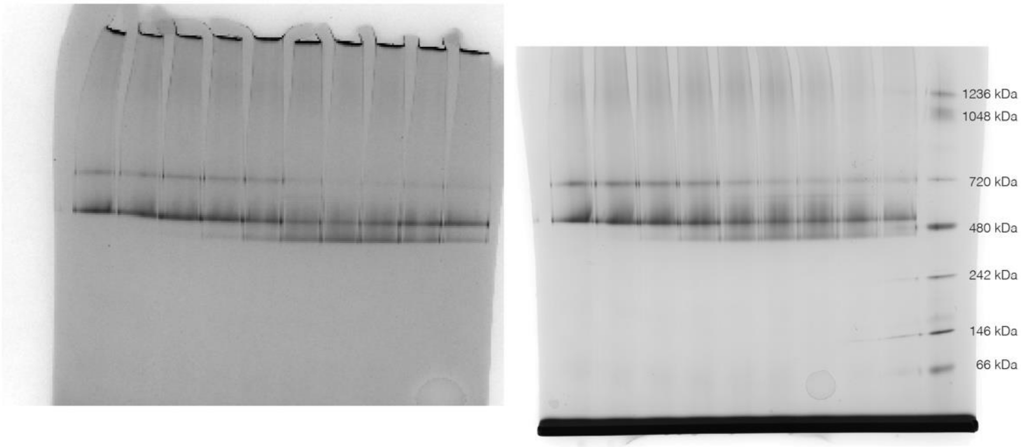

51

52 **Supplementary Figure 6 |** Two representative blue-native page gel repeats of Bcs1 as a function of AMP-PNP concentration.
53 Molecular weight (MW) marker on the right side of the second gel.

Supplementary Figure 7)

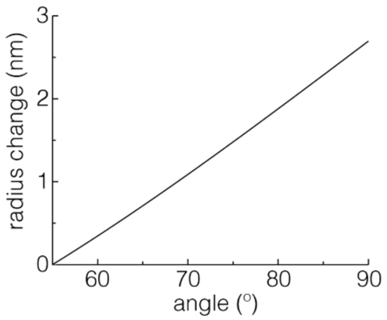

Supplementary Figure 7 | Radius change of the IMS pore gate formed by the Bcs1 TMHs as function of the TMH tilt angle α. α is the angle between the TMH long axis and the membrane plane (see Figure 5e in the main text). The pore is sealed at α = 55º as in the apo/ADP structures.

59 **Supplementary Figure 8)**

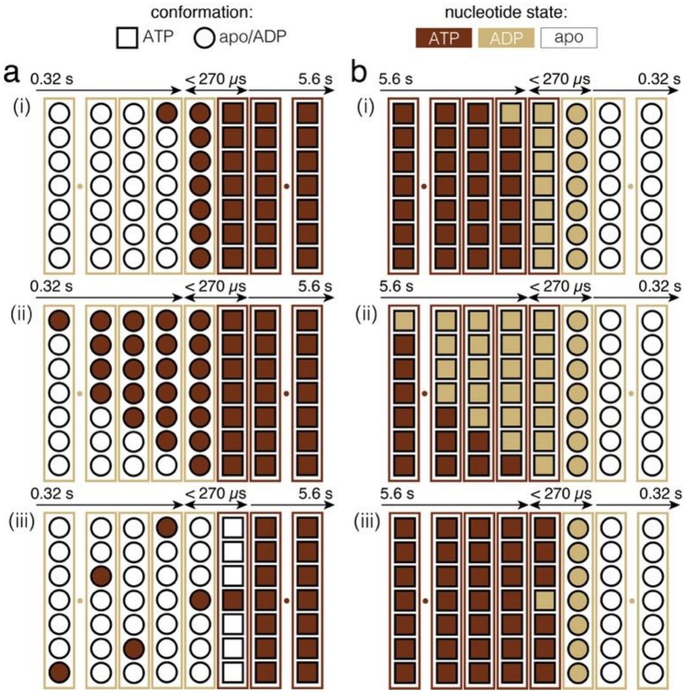

60

61 **Supplementary Figure 8 | Scenarios of coupling between conformational and nucleotide states in Bcs1. a)** Transition from
62 the apo/ADP- to ATP-conformation. **b)** Transition from the ATP- to apo/ADP -conformation.

Supplementary Figure 9)

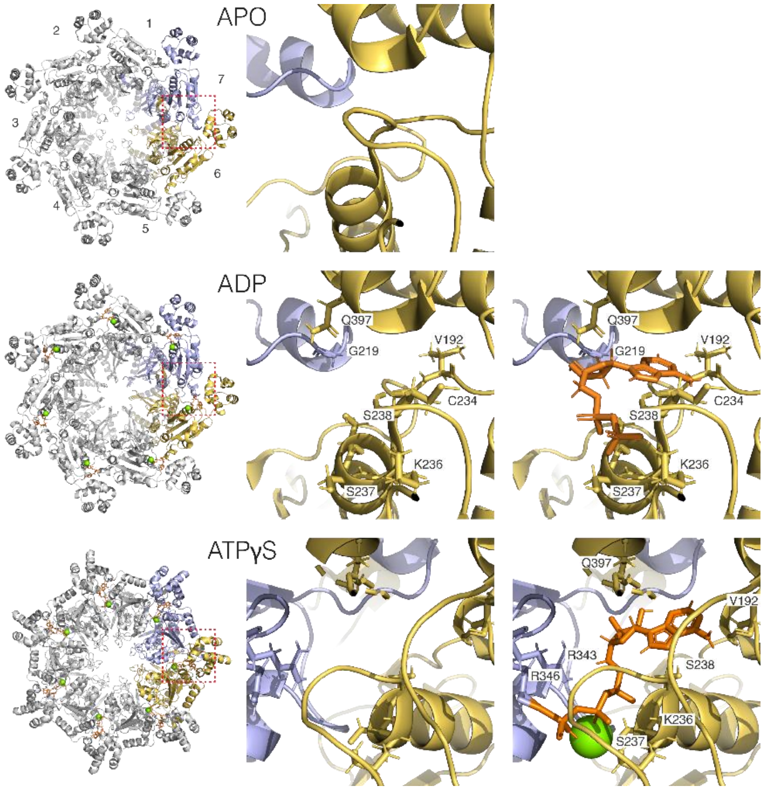

Supplementary Figure 9 | Nucleotide-binding pockets in apo, ADP and ATPγS Bcs1 structures. The apo and ADP conformations have similar binding sites (RMSD of ADP-interacting sidechains between apo and ADP conformation: 1.8 Å). The ATPγS-binding pocket is substantially different (RMSD ATPγS *vs.* apo: 10.1 Å; RMSD ATPγS *vs.* ADP: 10.5 Å). The binding pockets are located at the interface between two neighboring protomers (*middle*), involving G219 of protomer 1 (light blue), and G219 and V192, C234, K236, S237, S238 and Q397 of protomer 7 (yellow) for ADP, or involving R343 and R346 of protomer 1 (light blue), and Q397, V192, K236, S237 and S238 of protomer 7 (yellow) for ATPγS (*bottom*). (ADP and ATPγS are shown in orange).

Supplementary Figure 10)

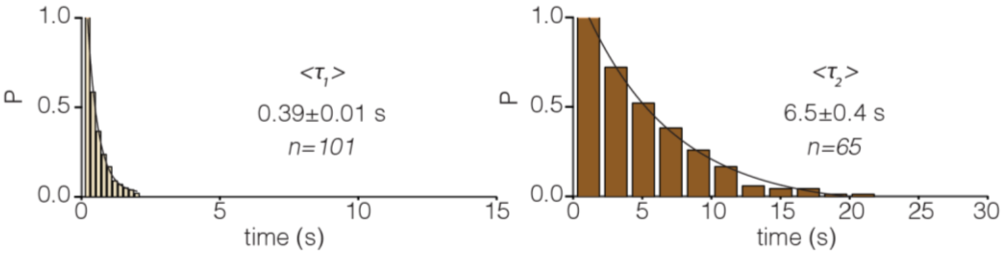

Supplementary Figure 10 | Dwell time distribution of the apo/ADP- (left) and ATP- (right) conformations in 10 µM ATP supplemented with 200 µM phosphate. The dwell time of the apo/ADP state is essentially unaffected by the presence of phosphate in the solution. Source data are provided as a source data file.

Supplementary Figure 11)

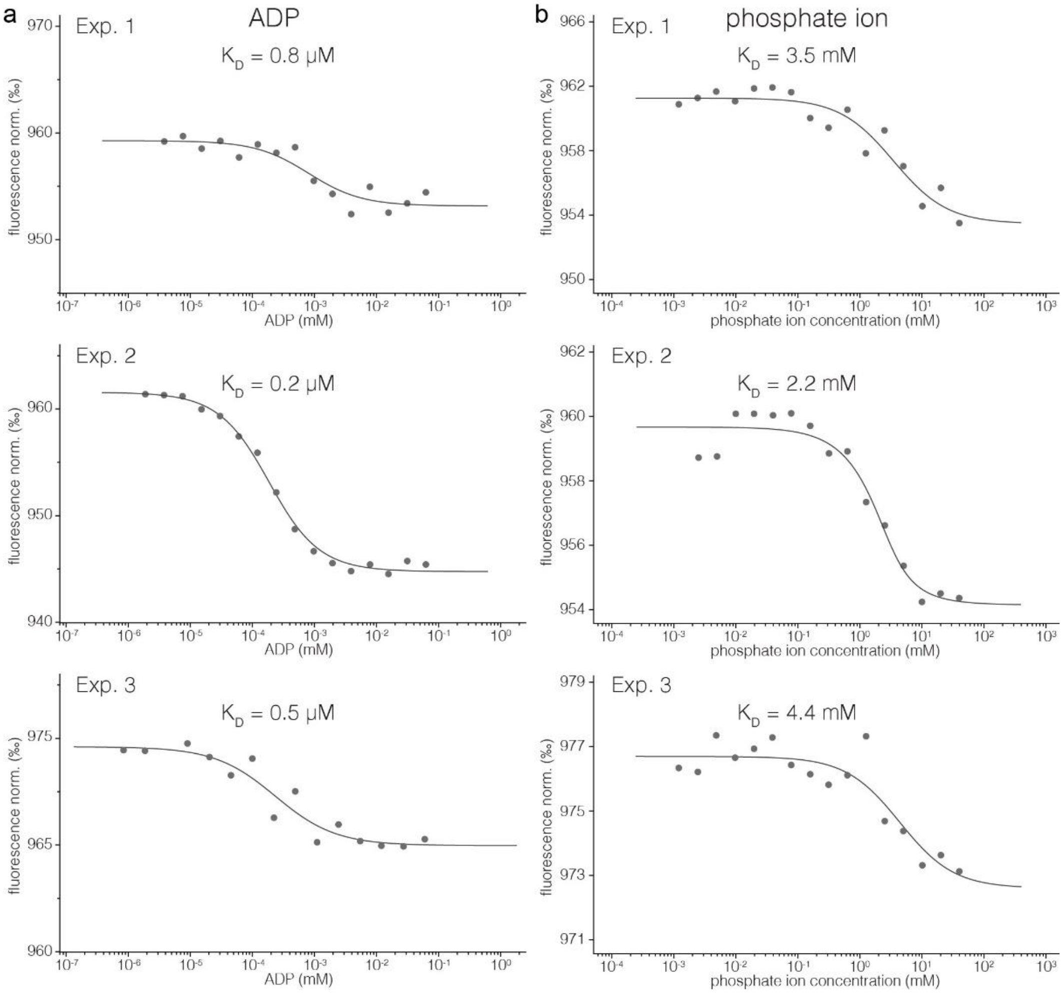

Supplementary Figure 11 | ADP-Bcs1 and P_i-Bcs1 binding studies using microscale thermophoresis (MST). a) Binding curves of fluorescence-labeled Bcs1 with ADP. The K_D value of ADP-Bcs1 is ~0.5 ± 0.3 µM. The error bars indicate *s.d.* of the mean value (n = 3). **b)** Binding curves of fluorescence-labeled Bcs1 with phosphate ions. The K_D value of P_i-Bcs1 is 3.4 ± mM. The error bars indicate *s.d.* of the mean value (n = 3). The concentration of fluorescence-labeled Bcs1 was kept constant (50 nM), and the concentration of ADP was varied from 0.00762 µM to 62.5 µM, and the concentration of phosphate ion was varied from 0.00122 mM to 40 mM. Source data are provided as a source data file.
