# Supplementary material for: A concerted ATPase cycle of the protein transporter AAA-ATPase Bcs1
Source: Nat Commun. 2023 Oct 11;14:6369. doi: 10.1038/s41467-023-41806-5 (PMC10567702; doi:10.1038/s41467-023-41806-5)
Supplement: Supplementary file 3 — Description of Additional Supplementary Files [file 41467_2023_41806_MOESM3_ESM.pdf]

Description of Additional Supplementary Files

Supplementary Movie 1
HS-AFM movie of apo-conformation Bcs1 IMS surface (See **Fig. 1d**)**.** The membranes appear as small periodic protrusions separated by extended membrane areas. Movie parameters: image size: 100 nm, frame size: 300 pixels, scan speed: 1 frame/s

Supplementary Movie 2
HS-AFM movie of apo conformation Bcs1 matrix surface (See **Fig. 1c,** *left*)**.** The membranes appear as ring structure. The seven subunits of the AAA-ATPase ring were clearly resolved. Movie parameters: image size: 60 nm, frame size: 300 pixels, scan speed: 1 frame/s

Supplementary Movie 3
HS-AFM movie of ADP conformation Bcs1 matrix surface (See **Fig. 1c,** *middle*)**.** HS-AFM imaging revealed that the ADP-conformational state Bcs1 has an overall similar conformation as apo-conformational state Bcs1 (see **Movie 2**). Movie parameters: image size: 60nm, frame size: 300 pixels, scan speed: 1 frame/s

Supplementary Movie 4
HS-AFM movie of ATPγS conformation Bcs1 matrix surface (See **Fig. 1c,** *right*)**.** HS-AFM imaging revealed that the ATPγS conformation Bcs1, instead of a ring structure, has a rather flat disk structure featuring seven peripheral protrusions. Movie parameters: image size: 100 nm, frame size: 300 pixels, scan speed: 1 frame/s

Supplementary Movie 5
HS-AFM movie of Bcs1 matrix surface conformational changes in presence of ATP (See **Fig. 2a**)**.** Bcs1 rings were observed switching back and forth between two conformations of different height and morphology. In the dark (low height) state, Bcs1 appears as relatively flat structure. In the bright (high height) state, Bcs1 appears as ring structure. Movie parameters: image size: 100 nm, frame size: 300 pixels, scan speed: 1 frame/s

Supplementary Movie 6
HS-AFM movie of Bcs1 matrix surface conformational changes in presence of ATP (high magnification) (See **Fig. 2d**)**.** Movie parameters: image size: 60 nm, frame size: 300 pixels, scan speed: 1 frame/s

Supplementary Movie 7
HS-AFM movie of Bcs1 matrix surface conformational changes in presence of ATP. Movie parameters: image size: 100 nm, frame size: 300 pixels, scan speed: 1 frame/s

Supplementary Movie 8
HS-AFM movie of Bcs1 IMS surface conformational changes in presence of ATP (See **Fig. 5a**)**.** The IMS side was observed switching dynamically between two conformations with different heights and widths. Movie parameters: image size: 100 nm, frame size: 300 pixels, scan speed: 1 frame/s
