# Supplementary material for: A concerted ATPase cycle of the protein transporter AAA-ATPase Bcs1
Source: Nat Commun. 2023 Oct 11;14:6369. doi: 10.1038/s41467-023-41806-5 (PMC10567702; doi:10.1038/s41467-023-41806-5)
Supplement: Supplementary file 12 — Reporting Summary [file 41467_2023_41806_MOESM12_ESM.pdf]

nature portfolio

Corresponding author(s): Simon Scheuring

Last updated by author(s): Sep 13, 2023

Reporting Summary

Nature Portfolio wishes to improve the reproducibility of the work that we publish. This form provides structure for consistency and transparency in reporting. For further information on Nature Portfolio policies, see our Editorial Policies and the Editorial Policy Checklist.

Statistics

For all statistical analyses, confirm that the following items are present in the figure legend, table legend, main text, or Methods section.

n/a	Confirmed	
☐	☒	The exact sample size (*n*) for each experimental group/condition, given as a discrete number and unit of measurement
☐	☒	A statement on whether measurements were taken from distinct samples or whether the same sample was measured repeatedly
☒	☐	The statistical test(s) used AND whether they are one- or two-sided<br>*Only common tests should be described solely by name; describe more complex techniques in the Methods section.*
☒	☐	A description of all covariates tested
☒	☐	A description of any assumptions or corrections, such as tests of normality and adjustment for multiple comparisons
☐	☒	A full description of the statistical parameters including central tendency (e.g. means) or other basic estimates (e.g. regression coefficient) AND variation (e.g. standard deviation) or associated estimates of uncertainty (e.g. confidence intervals)
☒	☐	For null hypothesis testing, the test statistic (e.g. *F*, *t*, *r*) with confidence intervals, effect sizes, degrees of freedom and *P* value noted<br>*Give P values as exact values whenever suitable.*
☒	☐	For Bayesian analysis, information on the choice of priors and Markov chain Monte Carlo settings
☒	☐	For hierarchical and complex designs, identification of the appropriate level for tests and full reporting of outcomes
☒	☐	Estimates of effect sizes (e.g. Cohen's *d*, Pearson's *r*), indicating how they were calculated

Our web collection on statistics for biologists contains articles on many of the points above.

Software and code

Policy information about availability of computer code

Data collection	Igor Pro 7 on HS-AFM (SS-NEX, RIBM, Tsukuba, Japan).
Data analysis	Matlab R2021a, ImageJ 1.53h, customized codes (avialibale from corresponding author upon reasonable request)

For manuscripts utilizing custom algorithms or software that are central to the research but not yet described in published literature, software must be made available to editors and reviewers. We strongly encourage code deposition in a community repository (e.g. GitHub). See the Nature Portfolio guidelines for submitting code & software for further information.

Data

Policy information about availability of data

All manuscripts must include a data availability statement. This statement should provide the following information, where applicable:

- Accession codes, unique identifiers, or web links for publicly available datasets
- A description of any restrictions on data availability
- For clinical datasets or third party data, please ensure that the statement adheres to our policy

The manuscript figures, supplementary figures supplementary movies, and source data files contain all data necessary to interpret, verify and extend the presented work. The raw AFM data is saved as .asd files and can only be opened using proprietary software. The raw data HS-AFM movies and customized codes for data analysis and simulation can be received from Simon Scheuring (sis2019@med.cornell.edu), upon reasonable request.

Human research participants

Policy information about studies involving human research participants and Sex and Gender in Research.

Reporting on sex and gender	N/A
Population characteristics	N/A
Recruitment	N/A
Ethics oversight	N/A

Note that full information on the approval of the study protocol must also be provided in the manuscript.

Field-specific reporting

Please select the one below that is the best fit for your research. If you are not sure, read the appropriate sections before making your selection.

☒ Life sciences ☐ Behavioural & social sciences ☐ Ecological, evolutionary & environmental sciences

For a reference copy of the document with all sections, see nature.com/documents/nr-reporting-summary-flat.pdf

Life sciences study design

All studies must disclose on these points even when the disclosure is negative.

Sample size	No statistical methods were used to determine the sample size. For HS-AFM movies of Bcs1, at least 97 frames for each conditions (APO, ADP, ATPγS, ATP) were acquired. For each frame, we analyzed at least 6 molecules, resulting in a dataset of at least 500 data points for the histogram, which proved sufficient for Gaussian fitting. For HS-AFM-LS, at least 78 (in most cases several hundreds events) are collected for generating the dwell-time distribution. This number is sufficient for the applied single exponential fitting. All the experiments were repeated at least 3 times.
Data exclusions	No data exclusions were made.
Replication	All the HS-AFM and HS-AFM-LS experiments were replicated at least 3 times.
Randomization	Randomization is not applicable to our biophysical experiments. This study did not allocate experimental/control groups.
Blinding	This is a molecular and structural biophysics study followed by computational data analysis-no blinding was required for this study. For the data analysis, we developed an objective analysis workflow, providing quantitative automated analysis of the collected data.

Reporting for specific materials, systems and methods

We require information from authors about some types of materials, experimental systems and methods used in many studies. Here, indicate whether each material, system or method listed is relevant to your study. If you are not sure if a list item applies to your research, read the appropriate section before selecting a response.

Materials & experimental systems

n/a	Involved in the study	
☒	☐	Antibodies
☒	☐	Eukaryotic cell lines
☒	☐	Palaeontology and archaeology
☒	☐	Animals and other organisms
☒	☐	Clinical data
☒	☐	Dual use research of concern

Methods

n/a	Involved in the study	
☒	☐	ChIP-seq
☒	☐	Flow cytometry
☒	☐	MRI-based neuroimaging
